# Architecting Durability: Synergies in Assembly, Self-Repair, and Advanced Characterization of Carbon Nanotube Materials

**DOI:** 10.3390/nano15171352

**Published:** 2025-09-02

**Authors:** Monika R. Snowdon, Shasvat Rathod, Robert L. F. Liang, Marina Freire-Gormaly

**Affiliations:** 1 Waterloo Institute for Nanotechnology, University of Waterloo, Waterloo, ON N2L 3G1, Canada; robliang@yorku.ca; 2 Lassonde School of Engineering, York University, North York, ON M3J 2S5, Canada; shasvat.rathod@uwaterloo.ca

**Keywords:** carbon nanotubes (CNTs), assembly, self-repair, durability, composites, advanced characterization, functionalization, spectroscopy

## Abstract

Carbon nanotubes (CNTs) have remarkable mechanical, electrical, and thermal properties, making them highly attractive as foundational elements for advanced materials. However, translating their nanoscale potential into macroscale reliability and longevity requires a holistic design approach that integrates precise architectural control with robust damage mitigation strategies. This review presents a synergistic perspective on enhancing the durability of CNT-based systems by critically examining the interplay between molecular assembly, self-repair mechanisms, and the advanced characterization techniques required for their validation. We first establish how foundational architectural control—achieved through strategies like chemical functionalization, field-directed alignment, and dispersion—governs the ultimate performance of CNT materials. A significant focus is placed on advanced functionalization, such as fluorination, and its verification using high-powered spectroscopic tools, including X-ray photoelectron spectroscopy (XPS) and near-edge X-ray absorption fine structure (NEXAFS) spectroscopy. Subsequently, this manuscript delves into the mechanisms of self-repair, systematically analyzing both the intrinsic capacity of the carbon lattice to heal atomic-level defects and the extrinsic strategies that incorporate engineered healing agents into composites. This discussion is uniquely supplemented by an exploration of the experimental techniques, such as electron energy loss spectroscopy (EELS) and Auger electron spectroscopy (AES), that provide crucial evidence for irradiation-induced healing dynamics. Finally, we argue that a “characterization gap” has limited the field’s progress and highlight the critical role of techniques like in situ Raman spectroscopy for quantitatively monitoring healing efficiency at the molecular level. By identifying current challenges and future research frontiers, this review underscores that the creation of truly durable materials depends on an integrated understanding of how to build, repair, and precisely measure CNT-based systems.

## 1. Introduction

### 1.1. The Durability Imperative in Advanced Carbon Materials

**Figure 1:**
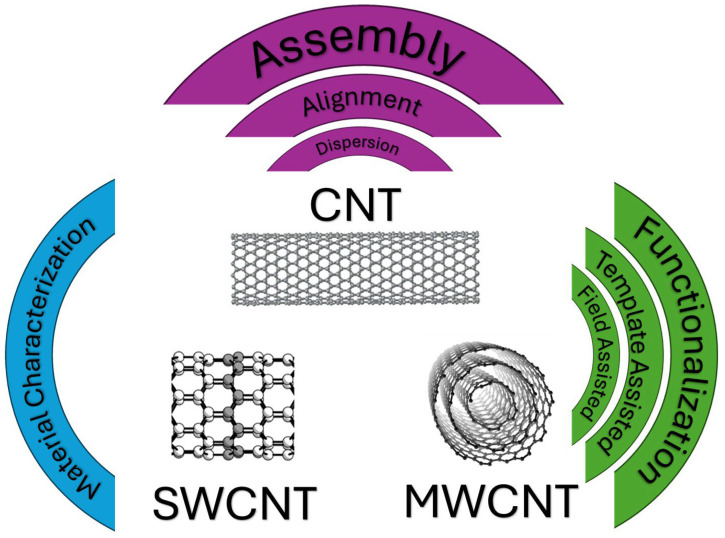
A schematic illustrating the fundamental concepts in engineering carbon nanotube (CNT) materials. The central image displays the basic molecular structure of a CNT, with its single-walled (SWCNT) and multi-walled (MWCNT) variants shown below. This is framed by the key engineering strategies discussed in this review: assembly (encompassing dispersion and alignment) and functionalization, which are critically validated through advanced material characterization.

Carbon nanotubes (CNTs), one-dimensional allotropes of carbon, have been the subject of intense scientific and technological interest due to their extraordinary combination of properties. These cylindrical nanostructures, which can be conceptualized as rolled-up sheets of graphene, exist as either single-walled (SWCNTs) or multi-walled (MWCNTs) as per Figure 1 and exhibit exceptionally high tensile strength, stiffness, electrical conductivity, and thermal conductivity [1]. These attributes make them ideal candidates for reinforcing polymers, metals, and ceramics, leading to the development of high-performance composites for demanding applications in aerospace, automotive, electronics, and energy storage [2,3,4]. However, the promise of these nanoscale properties is often unfulfilled in macroscopic applications due to a fundamental challenge: the accumulation of damage during service. Mechanical fatigue, environmental degradation, and operational stress can introduce defects, microcracks, and delamination, which compromise the structural integrity and functional performance of the material, ultimately leading to premature failure [4,5]. This reality has given rise to a “durability imperative” in materials science. It is no longer sufficient to design materials with high initial strength; they must be engineered to resist, tolerate, and even reverse damage over their intended lifespan. To this end, researchers have increasingly turned to nature for inspiration, where biological systems exhibit remarkable capabilities for autonomous damage repair, such as the healing of wounds or the regeneration of bone [2]. The translation of these biological principles to synthetic materials has led to the burgeoning field of self-repair, which aims to create materials that can autonomously detect and heal damage, thereby restoring their original properties and extending their service life. In the context of CNT-based systems, self-repair is not merely an intriguing feature but a critical design principle necessary to unlock their full potential for creating truly resilient and reliable next-generation materials.

### 1.2. State of the Art

The concepts of CNT assembly and self-healing materials are not new, and numerous reviews have capably summarized the progress in these individual areas [6]. The existing literature typically addresses topics such as assembly techniques (e.g., alignment, dispersion), self-healing mechanisms (e.g., microencapsulation, reversible bonds), and characterization methods as distinct and separate subjects. While valuable, this compartmentalized approach often overlooks the profound and synergistic relationships between these domains. The effectiveness of a self-repair mechanism is not an independent variable; it is fundamentally dependent on the initial material architecture established during assembly and the interfacial chemistry engineered through functionalization. A poorly dispersed network of CNTs, for instance, cannot provide the uniform conductive pathways necessary for efficient, thermally activated healing.

The novelty and innovation of this review lie in its integrated, synergistic framework. It posits that the design of durable CNT materials must be approached as a cohesive process, linking architecture to function. More critically, this review addresses a significant characterization gap that has emerged in the field. The maturation of self-healing materials research demands a shift from purely phenomenological observations of repair (e.g., a crack appears closed under a microscope) to a rigorous, quantitative understanding of the underlying molecular and atomic-level reconstruction. Conventional characterization methods, such as mechanical testing and standard microscopy, reveal the outcome of healing but are often insufficient to elucidate the process—the chemical rebonding, interfacial reconstruction, and atomic rearrangements that constitute true repair.

Therefore, this review provides a unique contribution by being the first to comprehensively bridge the design of self-healing CNT materials with the advanced analytical toolkit required to validate their function at a fundamental level. It will build a logical argument that flows from foundational chemical functionalization and physical assembly, through a detailed examination of intrinsic and extrinsic repair mechanisms, to a critical discussion of the high-powered spectroscopic techniques (e.g., XPS, NEXAFS, EELS, Auger, and in situ Raman) that are essential for probing and proving the efficacy of these processes. By framing the discussion in this manner, this work transforms from a descriptive summary into a prescriptive guide for the rigorous design and analysis of the next generation of durable, intelligent materials.

## 2. Foundational Architectures: Controlled Assembly and Functionalization of CNTs

The macroscopic performance of any CNT-based material is dictated by the precise arrangement and interaction of its nanoscale constituents. This section reviews the critical parameters of CNT assembly—alignment, dispersion, and density—and introduces advanced functionalization as a foundational strategy to enable this architectural control.

### 2.1. Impact of Alignment on Mechanical, Electrical, and Thermal Properties

The alignment of CNTs within a matrix is a pivotal factor in transitioning their anisotropic nanoscale properties to the macroscale. In terms of electrical properties, aligned CNTs create highly efficient, direct pathways for electron transport. This dramatically lowers the electrical percolation threshold—the minimum concentration of conductive filler required to form a connected network [7]. For example, studies on CNT–epoxy composites have shown that aligning the nanotubes can reduce the percolation threshold by more than an order of magnitude, from approximately 0.034 vol% for randomly oriented CNTs to as low as 0.0031 vol% when measured parallel to the alignment direction [8]. This structural precision is a significant advancement for applications in miniaturized electronics, as it allows for the same or better conductivity to be achieved with over 90% less CNT content [8,9].

**Figure 2:**
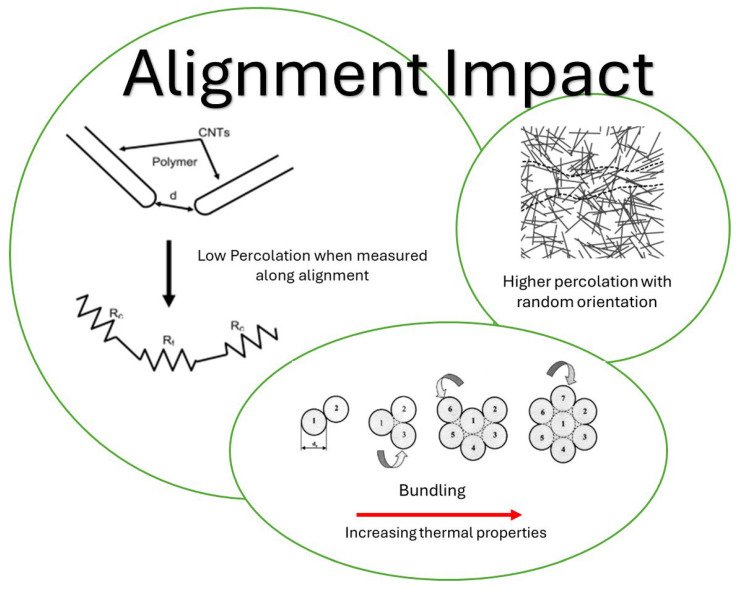
Illustration of alignment impact on CNT materials. Adapted from [12,13].

This architectural control simultaneously enhances mechanical robustness. When aligned, CNTs act as reinforcing fibers, effectively bearing a significant portion of any applied load and hindering the propagation of cracks [10,11]. This leads to measurable improvements in both the elastic modulus and fracture toughness of the composite. The aligned nanotubes force cracks to deviate and follow more tortuous paths, dissipating more energy and increasing the material’s resistance to failure [12]. Similarly, thermal conductivity is greatly improved along the direction of alignment, as the ordered structure provides efficient pathways for phonon transport, which is critical for thermal management applications requiring effective heat dissipation [12,13]. Conversely, misalignment leads to increased phonon scattering at nanotube–nanotube interfaces, reducing overall thermal conductivity (see Figure 2) [14].

### 2.2. Influence of Dispersion on Material Characteristics

Achieving a uniform dispersion of CNTs within a matrix material is fundamental to harnessing their full potential. Due to strong van der Waals forces and π−π stacking interactions, CNTs have a natural tendency to agglomerate into bundles [15]. These agglomerates act as defects within the composite, creating stress concentration points that can initiate premature failure under a mechanical load and disrupting the formation of continuous networks required for electrical and thermal conductivity [12]. Poor dispersion has been directly linked to reduced mechanical strength, toughness, and conductivity in composites [16,17]. For instance, while well-dispersed CNTs can form a reinforcing meshwork in materials like cement mortar, inadequate dispersion can lead to increased porosity and the creation of weak zones, ultimately diminishing the material’s mechanical integrity [7,18]. A homogeneous distribution ensures effective load transfer from the matrix to the high-strength nanotubes and allows for the formation of an efficient, interconnected conductive network at lower CNT concentrations [15]. 

### 2.3. Effects of Density on Material Performance

The density of CNTs, both in their individual form and in bulk assemblies, is another key parameter governing material performance [19]. The inherently low density of individual CNTs is a primary reason for their exceptionally high specific strength (strength-to-weight ratio), which far surpasses that of traditional materials like high-carbon steel [1]. This characteristic is particularly advantageous in weight-sensitive industries such as aerospace and automotive. At the bulk level, the packing density of CNT assemblies significantly influences their collective properties [20]. For instance, research has shown that the specific thermal conductivity of CNT films and fibers tends to decrease as the bulk density increases, highlighting the complex interplay between individual tube properties and their collective arrangement. Advanced synthesis techniques, such as plasma-enhanced chemical vapor deposition (PECVD), allow for precise control over CNT density by modulating catalyst density and growth conditions [21,22]. This tunability is essential for optimizing performance in specific applications, such as thermal interface materials that require high conductivity at specific densities or field emitters where the density of emission sites is a critical parameter [17,23,24].

### 2.4. Challenges in Achieving Uniform and Scalable Assembly

Despite significant progress, achieving uniform and scalable assembly of CNTs remains a major hurdle. The primary challenge stems from the strong intermolecular forces that cause CNTs to aggregate, making homogeneous dispersion difficult to achieve and maintain [1]. Overcoming these forces is a prerequisite for creating high-performance composites. Furthermore, many laboratory-scale techniques that produce highly ordered CNT structures, such as chemical vapor deposition (CVD) and electrophoresis, face significant challenges when scaling up for industrial production. These methods often require stringent control over process parameters that are difficult to maintain over large areas, rendering them costly and limiting their throughput [25]. The inherent variability in CNTs produced by common synthesis methods, which yield a mixture of diameters and chiralities, further complicates uniform assembly and leads to inconsistencies in the final material’s properties [1]. Finally, residual catalyst nanoparticles and amorphous carbon impurities from the synthesis process can contaminate the CNTs, negatively affecting their properties and requiring purification steps that can themselves introduce structural damage [26].

### 2.5. Advanced Functionalization for Enhanced Interfacial Engineering

The challenges in achieving uniform dispersion and assembly are fundamentally rooted in the poor interfacial compatibility between the pristine, hydrophobic surfaces of CNTs and many host matrices, particularly polymers. Chemical functionalization is a powerful strategy to overcome this barrier by chemically modifying the CNT surface to improve its compatibility, thereby enabling more effective assembly [27]. This process is not merely an optional enhancement but often a necessary prerequisite for creating the well-defined architectures discussed previously.

#### 2.5.1. Covalent Functionalization: The Case of Fluorination

Among various covalent functionalization strategies, direct fluorination has emerged as a well-established and highly effective method for altering the surface properties of CNTs [28]. The process involves treating CNTs with gaseous fluorine, often at elevated temperatures, to form covalent C-F bonds on the nanotube sidewalls. This reaction changes the hybridization of the carbon atoms at the bonding sites from graphitic sp^2^ to diamond-like sp^3^. The degree of fluorination can be controlled by tuning reaction conditions such as temperature and fluorine concentration, allowing for the synthesis of materials with a desired stoichiometry, such as CFx, where x can be tuned up to ~0.5 while preserving the core tubular structure of the nanotube [18,28,29].

The covalent attachment of fluorine atoms dramatically alters the properties of the CNTs. The introduction of polar C-F bonds significantly increases the hydrophilicity of the nanotubes, rendering them soluble or dispersible in various solvents, including alcohols and water, which is a major advantage of solution-based processing and integration into polymer matrices [28]. This enhanced compatibility directly addresses the challenge of agglomeration, facilitating the creation of the uniform dispersions that are critical for achieving optimal mechanical and electrical properties in the final composite.

#### 2.5.2. Spectroscopic Verification of Surface Chemistry: XPS, NEXAFS, and SXE Analysis

Verifying the success and nature of the chemical modification is a critical step in any functionalization process. A suite of high-powered, surface-sensitive spectroscopic techniques is essential for this task, providing detailed information on the elemental composition and electronic structure of the modified CNTs.

X-ray Photoelectron Spectroscopy (XPS) is a cornerstone technique for surface chemical analysis. By irradiating a sample with X-rays and analyzing the kinetic energy of the emitted photoelectrons, XPS provides quantitative information about the elemental composition and the chemical bonding environment of the near-surface region (~10 nm depth) [30]. In the case of fluorinated CNTs, XPS can unambiguously confirm the presence of fluorine and, through high-resolution analysis of the C 1s and F 1s core-level spectra, can identify the formation of covalent C-F bonds and distinguish them from non-covalently adsorbed fluorine species [28,31].

Near-Edge X-ray Absorption Fine Structure (NEXAFS) Spectroscopy provides complementary information by probing the unoccupied electronic states of the material. This technique is particularly sensitive to the local bonding environment and hybridization of carbon atoms. In pristine CNTs, the NEXAFS spectrum is characterized by a sharp peak corresponding to transitions into unoccupied π∗ orbitals, a hallmark of sp^2^ hybridization. Upon covalent fluorination, the intensity of this π∗ peak decreases, and new features corresponding to σ∗ orbitals associated with sp^3^ C-F bonds appear [32]. Studies on fluorinated MWCNTs using NEXAFS have confirmed the formation of covalent C-F bonds and demonstrated that, under optimal conditions, the fluorination process can be uniform throughout the probing depth of the technique (~15 nm) [31,33].

Soft X-ray Emission (SXE) Spectroscopy completes the electronic structural characterization by probing the occupied electronic states (the valence band). When used in conjunction with NEXAFS, which probes the unoccupied states (the conduction band), SXE provides a comprehensive picture of how functionalization modifies the entire electronic band structure of the CNTs. The successful application of these techniques is detailed in the literature, for example, in the works of Krestinin et al. and Brzhezinskaya et al., which provide foundational studies on the production and advanced spectroscopic characterization of fluorinated single-walled and multi-walled CNTs, respectively [28,31].

## 3. Mechanisms and Analysis of Self-Repair in CNT-Based Systems

The ability of a material to autonomously heal damage is a transformative property that can dramatically enhance its durability and reliability. This chapter explores the fundamental mechanisms of self-repair in the context of CNT-based materials, beginning with the inherent repair capabilities of the carbon lattice itself, followed by engineered extrinsic strategies. Critically, it also details the advanced analytical tools required to observe and quantify these healing processes at the molecular level [34,35].

### 3.1. Intrinsic Repair: Atomic-Level Healing in Carbon Lattices

Intrinsic self-repair refers to the inherent ability of a material’s structure to heal defects without the need for external healing agents. In CNTs, this capability is rooted in the dynamic nature of the carbon lattice under certain conditions.

#### 3.1.1. Defect Annihilation and Bond Reconfiguration

The carbon atoms in a CNT lattice are not static. When provided with sufficient activation energy, they can rearrange to form more stable configurations. One of the most well-known mechanisms for this is the Stone–Wales transformation, a 90-degree bond rotation that can create or, more importantly for healing, annihilate topological defects like pentagon–heptagon pairs within the hexagonal carbon lattice. Beyond topological defects, the lattice can also heal vacancies and other structural imperfections. This process is driven by external stimuli that provide the necessary energy for atomic migration and bond reconfiguration. High temperatures can supply the thermal energy for atoms to move and fill vacancies, effectively annealing out defects. Similarly, irradiation with electron or ion beams can both induce defect formation and, under controlled conditions, facilitate healing by promoting atomic rearrangement. The local chemical environment can also play a role, for example, by providing atoms to fill vacancies or by altering the energy barriers for atomic rearrangements. However, these intrinsic mechanisms are typically effective only for atomic-scale damage and have limitations when faced with larger cracks or harsh operating conditions.

#### 3.1.2. Experimental Probes of Irradiation-Induced Healing: Insights from EELS and Auger Spectroscopy

While theoretical simulations provide valuable insights into atomic-level healing, experimental verification is crucial. Ion irradiation serves as a powerful tool for this purpose, acting dually as a method to controllably introduce defects for study and as an energy source to potentially trigger healing processes. The atomic-scale structural and electronic changes resulting from this process can be probed with high-resolution spectroscopic techniques. Electron energy loss spectroscopy (EELS), typically performed in a scanning transmission electron microscope (STEM), offers the ability to acquire chemical and electronic information with sub-nanometer spatial resolution [36,37]. EELS spectra from carbonaceous materials exhibit distinct features, including a π−π∗ plasmon peak at lower energy losses, which is characteristic of sp2-bonded carbon, and a higher-energy π+σ plasmon peak [38]. The fine structure of the carbon K-edge ionization loss provides detailed information on the unoccupied density of states, allowing for the quantification of the sp^2^/sp^3^ bonding ratio [38]. Ion irradiation induces damage that disrupts the sp^2^ lattice, leading to a decrease in the energy and intensity of the π plasmon and changes in the C K-edge fine structure [38,39]. By monitoring these spectral features as a function of irradiation dose and other conditions (e.g., temperature), EELS can provide direct experimental evidence of the dynamics of defect formation and subsequent atomic-level healing or structural rearrangement. Auger electron spectroscopy (AES) is a complementary, highly surface-sensitive technique that provides information on the elemental composition and chemical bonding environment of the top few atomic layers of a material. The kinetic energy and, more importantly, the shape of the C KVV Auger transition are highly sensitive to the local chemical environment and hybridization of the carbon atoms. The C KVV lineshape serves as a “fingerprint” that can distinguish between graphitic (sp^2^), diamond-like (sp^3^), and amorphous carbon [39,40]. Consequently, AES can be used to characterize the nature of the CNT surface before and after ion irradiation. Changes in the C KVV lineshape can reveal the transformation from a well-ordered graphitic structure to a more disordered or sp^3^-rich state upon damage, and any subsequent recovery of the graphitic signature would provide evidence of intrinsic healing [41]. These experimental approaches, as detailed in studies such as those investigating π-plasmons in ion-irradiated nanotubes, provide the crucial empirical data needed to validate and understand the fundamental mechanisms of intrinsic repair [39].

### 3.2. Extrinsic Repair: Engineering Healing into the Composite

Extrinsic self-repair involves incorporating external healing functionalities into a material that does not possess an inherent healing capacity. This is typically achieved by embedding reservoirs of healing agents within the composite matrix.

#### 3.2.1. Reservoir-Based Healing: Microcapsules and Vascular Networks

The most common extrinsic approach is microencapsulation [1]. In this method, a liquid healing agent, such as the monomer dicyclopentadiene (DCPD), is encapsulated within small, brittle-shelled microcapsules such as urea-formaldehyde. These microcapsules are then dispersed throughout the composite matrix along with a corresponding catalyst, including Grubbs’ catalyst [13,42,43]. When a crack propagates through the material, it ruptures the embedded microcapsules, releasing the healing agent into the crack plane via capillary action [44]. The monomer then comes into contact with the dispersed catalyst, initiating a polymerization reaction that bonds the crack faces together, thereby restoring structural integrity [42]. For healing chemistries that require two separate components (e.g., epoxy and a hardener), a dual-microcapsule system can be employed, where each component is encapsulated separately. A more advanced, bio-inspired strategy utilizes vascular networks. These systems consist of interconnected microchannels or hollow fibers embedded within the composite, mimicking biological circulatory systems [45]. These networks can be filled with a healing agent, which is delivered to a damaged site when the network is breached by a crack. The primary advantage of vascular networks over microcapsules is their potential for repeated healing. While microcapsules are a finite resource that is depleted after a single rupture event, vascular networks can be refilled from an external reservoir, allowing for multiple healing cycles at the same damage location [45]. 

#### 3.2.2. The Multifaceted Role of CNTs in Matrix Reconstruction

Carbon nanotubes can be integrated into extrinsic self-healing systems to play several crucial roles that enhance the repair process and the properties of the healed material [45]. Their contribution goes beyond simple reinforcement and can be tailored to enable more advanced, stimulus-responsive healing mechanisms. First, CNTs serve as potent mechanical reinforcement for the healed region [3,46]. When dispersed within the healing agent (e.g., co-encapsulated within microcapsules), they are delivered to the crack plane along with the monomer. After polymerization, the CNTs are embedded within the newly formed polymer, where they can bridge the crack faces and improve the load transfer across the repaired interface, leading to a higher recovery of mechanical properties like strength and toughness [3,46]. Second, the hollow core of CNTs can be utilized as nanoscale healing agent reservoirs. In this concept, the nanotubes themselves are filled with a healing agent and dispersed in the matrix. Damage that causes the nanotubes to rupture would then trigger the release of the encapsulated agent to initiate repair. Third, and perhaps most significantly, a percolated network of CNTs within a composite matrix can enable stimulus-responsive healing via Joule heating [47,48]. When an electric current is passed through the conductive CNT network, resistance heating (Joule heating) generates thermal energy. This can be used to trigger self-repair in several ways [47,48]. In a thermoplastic matrix, the localized heat can melt the polymer in the vicinity of the crack, allowing it to flow, fill the void, and solidify upon cooling [47,48]. In thermoset matrices incorporating reversible chemical bonds (e.g., Diels–Alder adducts), the heat can provide the activation energy needed to break and reform these bonds across the crack interface, effectively re-welding the material. Finally, the high surface area and unique electronic properties of CNTs give them the potential to act as catalysts for the polymerization of certain healing agents, potentially eliminating the need for a separate, dispersed catalyst and simplifying the material system.

### 3.3. A Modern Toolkit for Characterizing Self-Repair Efficiency

Quantifying the efficiency of a self-repair process requires a multi-faceted characterization approach that can assess the restoration of properties from the macroscopic to the molecular scale.

#### 3.3.1. Mechanical and Morphological Restoration

The most common method for evaluating healing efficiency is through destructive mechanical testing. Techniques such as tensile tests, fracture toughness tests, and fatigue tests are used to compare the mechanical properties of a pristine material with those of a sample that has been damaged and subsequently healed [49,50]. The healing efficiency is typically reported as the percentage of the original property that is recovered [3,46,49,50]. These tests provide a critical measure of the functional restoration of the material. Complementing this, microscopy techniques like scanning electron microscopy (SEM) and atomic force microscopy (AFM) are used for visual assessment. These methods can directly image the damaged area before and after healing, providing qualitative and quantitative information on crack closure and the morphology of the healed region [49,50]. 

#### 3.3.2. In Situ Monitoring of Healing Dynamics with Raman Spectroscopy

Raman spectroscopy is a non-destructive optical technique that provides detailed information about the vibrational modes of molecules, making it exceptionally well-suited for studying the molecular structure of carbon materials [8,17,51,52,53,54]. The Raman spectrum of CNTs is characterized by several key features: the G-band (~1582 cm^−1^), which arises from the in-plane bond stretching of sp^2^ carbon atoms and is indicative of the graphitic nature of the nanotube; the D-band (~1350 cm^−1^), which is activated by defects, disorder, or the edges of the graphene lattice; and the 2D (or G’) band (~2700 cm^−1^), which is an overtone of the D-band [51,52,53,54]. The intensity ratio of the D-band to the G-band (ID/IG) is a widely used and well-established metric for the density of defects in the carbon lattice. This relationship provides a powerful avenue for quantitatively monitoring self-healing. Damage to a CNT-based composite, such as a crack, inherently introduces disorder and breaks bonds, leading to a localized increase in the ID/IG ratio [8,17,51,52,53,54]. A successful intrinsic healing event, which involves the reconstruction of the graphitic lattice, should reduce this disorder, resulting in a decrease in the ID/IG ratio. This allows for the development of a quantitative, spectroscopic measure of healing efficiency. By using Raman mapping to scan the damaged area before and after a healing cycle, one can generate a spatially resolved map of the defect density [51,52,53,54]. The reduction in the integrated ID/IG ratio across the healed region provides direct, molecular-level evidence of structural repair. For extrinsic systems where healing involves polymerization, Raman spectroscopy can be used in situ to monitor the reaction kinetics by tracking the disappearance of monomer-specific vibrational peaks and the appearance of polymer peaks [8,17].

#### 3.3.3. Interfacial Chemistry in Repaired Zones via High-Powered Spectroscopy

While mechanical tests and Raman spectroscopy can confirm that healing has occurred, a deeper understanding requires probing the chemical nature of the newly formed interface within the repaired zone. The advanced spectroscopic techniques discussed earlier for functionalization analysis are also invaluable for post-mortem analysis of healed materials. After a sample has been damaged and healed, techniques like NEXAFS, EELS, AES, and SXE can be employed to analyze the chemical composition and bonding across the healed crack plane [37]. For instance, these methods can verify that an extrinsic healing agent has successfully formed covalent bonds with the original matrix material, or they can characterize the electronic structure of an intrinsically healed region to confirm the restoration of the graphitic lattice. This level of detailed chemical information is critical for optimizing healing chemistries and understanding failure mechanisms in healed materials, as demonstrated in studies of polymer–nanocarbon composite interfaces [55].

To synthesize and clarify the roles of these advanced techniques, Table 1 provides a comparative overview of their capabilities in the context of self-healing analysis.

## 4. Synergistic Effects of Molecular Assembly and Self-Repair: Influence of CNT Assembly

The efficiency and effectiveness of a self-repair mechanism are not isolated properties but are intimately linked to the underlying architecture of the CNT network within the material. The deliberate control over CNT assembly, as discussed in Section 2, creates foundational structures that can be leveraged to enhance the transport of healing agents, the accessibility of damage sites, and the overall durability of the composite.

### 4.1. Impact of CNT Alignment on Healing Agent Transport and Diffusion

The assembly of CNTs into ordered architectures, particularly through alignment, offers a promising strategy for enhancing the efficiency of self-repair mechanisms in advanced materials. A key advantage of aligned CNT networks is their capacity to serve as nanoscale conduits for the transport and diffusion of healing agents to sites of material damage. Research in water purification has shown that aligned and encapsulated CNTs with open ends facilitate the flow of water molecules through their interiors [56]. This inherent nanoscale fluid transport capability suggests that aligned CNTs can similarly function as efficient pathways for the delivery of liquid healing agents within a material matrix. Directional alignment enables a more targeted and rapid delivery of these agents compared to the slower, isotropic diffusion characteristic of bulk material matrices. Furthermore, the diameter of CNTs can be tailored to control the transport of specific healing agents based on molecular size, offering an additional degree of control over the self-repair process. The role of CNT alignment in guiding biological entities is also well-documented. For instance, aligned CNT yarn-patterned substrates have been shown to act as topographic scaffolds that direct neurite outgrowth. This demonstrates that the directional guidance provided by aligned CNTs extends beyond fluid transport to influence the movement and growth of micro- and nanoscale entities [57]. In the context of self-repair, this suggests that aligned CNTs might not only facilitate the transport of healing agents but could also guide their subsequent reaction or polymerization at the damage site, leading to a more structured and effective repair. The foundational principle is that uniaxial alignment, whether achieved by hot rubbing, blade coating, or gas flow, enhances directional transport properties parallel to the orientation. The development of advanced deposition techniques, such as those utilizing a vapor-mediated Marangoni effect to create locally aligned networks, further expands the toolkit for creating these functional architectures [58,59]. Moreover, the effectiveness of these aligned networks is predicated on starting with high-purity, single-chirality nanotubes, which can be isolated using advanced, scalable sorting methods like salt-driven aqueous two-phase extraction [60]. This demonstrates that the directional guidance provided by aligned CNTs extends beyond fluid transport to influence the movement and growth of micro- and nanoscale entities. In the context of self-repair, this suggests that aligned CNTs might not only facilitate the transport of healing agents but could also guide their subsequent reaction or polymerization at the damage site, leading to a more structured and effective repair. The mechanical properties of CNT-based materials are strongly influenced by the degree of CNT alignment.

For CNT fibers, tensile strength is closely related to the orientation of constituent CNTs. Achieving a high degree of alignment—often through processes such as wet-spinning followed by drafting and thermal annealing—is critical for maximizing mechanical performance [61]. Similarly, for efficient healing agent transport, a well-aligned and densely packed network of CNTs is essential to provide continuous and unobstructed pathways to the damage. Imperfect alignment can result in tortuous routes and reduced transport efficiency [61]. CNT alignment also plays a critical role in enhancing the efficiency of stimulus-based self-healing mechanisms, particularly those involving Joule heating [48]. By creating efficient conductive pathways, aligned CNTs allow for localized heat generation when an electric field is applied. This heat can activate thermally responsive self-healing mechanisms in the surrounding material. Alignment ensures that electrical current flows more readily through the network, leading to targeted and effective delivery of thermal energy to the damage site compared to randomly dispersed CNT networks [48]. The consistent improvement in performance observed with aligned CNTs across various applications—from fluid transport and biological guidance to enhanced electrical and thermal properties—highlights the fundamental importance of controlling CNT orientation for optimizing material functionalities relevant to self-repair. This control can influence both the direct transport of healing agents through CNT lumens and the material’s response to external stimuli used to activate healing mechanisms.

### 4.2. Role of CNT Dispersion in Damage Site Accessibility for Self-Repair Mechanisms

As discussed in Section 2, beyond alignment, the dispersion of CNTs within a material matrix is another critical factor influencing self-repair efficiency, particularly with respect to the accessibility of damage sites for healing agents and mechanisms [62]. Microencapsulation strategies, in which CNTs are suspended within a self-healing monomer, illustrate the importance of dispersion. Uniform distribution of CNTs within the healing agent reservoir ensures that nanoparticles are effectively delivered to the damage site upon capsule rupture. This intimate mixing allows CNTs to reinforce the repaired area or facilitate healing reactions at the crack interface. Poor dispersion can lead to CNT agglomeration, resulting in uneven distribution within the released healing agent and localized property enhancement rather than uniform improvement across the healed region [62]. Research has directly linked the quality of CNT dispersion to the effectiveness of self-healing mechanisms, especially those involving Joule heating [48,62]. A homogeneous dispersion of CNTs leads to a more uniform conductive network throughout the material, which is essential for generating heat evenly across the damaged area when an electric current is applied. This ensures that the entire crack interface experiences the necessary temperature for efficient healing. For example, in composites designed for thermal healing, well-dispersed CNTs act as efficient heat conductors that enable the rapid transfer of thermal energy to a healing agent like polycaprolactone (PCL), accelerating its transport into damaged areas [63].

Conversely, poor dispersion can result in localized CNT concentrations, leading to uneven heating and potentially incomplete or inefficient repair in areas with fewer nanotubes. The importance of dispersion is also highlighted in repair techniques for composite laminates, where functionalized CNTs with improved dispersibility can be transported deep into microcracks via the capillary action of a solvent carrier, ensuring the reinforcing agent reaches the damage site [64]. The dispersibility of CNTs is also influenced by their physical characteristics, such as length. Shorter CNTs tend to disperse more readily in a matrix compared to longer ones, which are more prone to entanglement and aggregation due to increased van der Waals forces. While longer CNTs may be desirable for reinforcement due to their higher aspect ratio, improved dispersion of shorter CNTs enhances damage site accessibility by ensuring a more homogeneous distribution throughout the material. The impact of CNT dispersion on mechanical properties further underscores its importance for self-repair. Properly dispersed CNTs improve load transfer efficiency within a composite material, whereas poor dispersion leading to agglomerated clusters can restrict stress distribution and weaken mechanical properties. Since self-repair aims to restore mechanical integrity, good CNT dispersion is crucial for successful repair. Agglomerated CNTs can act as stress concentration points, potentially leading to re-cracking or incomplete restoration of strength after healing [62]. Achieving uniform and stable CNT dispersion often requires tailored strategies based on the properties of both CNTs and the matrix material. Surface functionalization of CNTs can improve their compatibility with polymer matrices, enhancing dispersibility in aqueous or organic solvents. Specialized assembly techniques, such as electrostatic self-assembly, have been used to promote uniform CNT distribution in materials like cement paste [65]. These approaches highlight the need for careful consideration of interfacial interactions between CNTs and the surrounding matrix to achieve optimal dispersion for effective self-repair.

### 4.3. Demonstrations of Enhanced Durability Through Combined Approaches

The literature provides numerous compelling case studies that demonstrate the successful integration of carbon nanotube assembly with various self-repair mechanisms, leading to significant enhancements in material durability, lifespan, and healing efficiency. These studies collectively highlight the versatility of CNTs and the synergistic effects achieved by precisely controlling their architecture within different material systems. Table 2 provides a summary of representative case studies.

These studies illustrate a variety of approaches, including the use of dispersed CNTs to enable Joule heating for thermally activated healing [50], the assembly of CNTs into specific architectures like interleaves for targeted repair in composites [67], and the utilization of the unique structure of CNTs for healing agent storage and delivery [35]. Furthermore, the dynamic assembly of dispersed CNTs in response to an electric field offers a novel solution for self-repair in electronic circuits [68]. The consistent theme across these studies is that by carefully controlling the assembly of CNTs within a material, it is possible to significantly enhance its ability to autonomously or through external stimuli repair damage, leading to improved durability and an extended lifespan.

## 5. Challenges and Future Opportunities in Self-Healing CNT Materials

### 5.1. Advanced Characterization Techniques for Studying Self-Healing Processes at Different Scales

While mechanical characterization techniques effectively quantify the recovery of mechanical properties, specialized methods are needed to assess the restoration of other crucial functionalities, such as electrical and thermal conductivity, as discussed in Section 3.3, are paramount in many CNT applications. Developing techniques to measure these properties in situ during healing and after repeated cycles would offer a more complete evaluation of the self-healing efficiency. Complementing these experimental methods, advanced computational approaches like ReaxFF molecular dynamics and density functional theory (DFT) are critical for investigating microscopic mechanisms that are difficult to observe directly [72]. Such simulations can provide atomic-level insights into the structural evolution of CNTs at elevated temperatures, including defect formation, bond rearrangement, and the transformation of end-cap structures, which are fundamental to understanding intrinsic repair and material stability during thermal healing cycles [72].

Characterizing the interface between CNTs and the matrix remains a significant challenge. Advanced techniques like high-resolution electron microscopy with energy-dispersive X-ray spectroscopy (EDS) or focused ion beam (FIB) milling combined with SEM could provide more detailed information about the interfacial structure and bonding, and how these are affected by damage and repair [73].

Finally, the establishment of standardized protocols and metrics for characterizing self-healing in CNT materials is crucial for ensuring reproducibility and comparability across different research studies. This includes defining clear methods for inducing damage, quantifying the extent of healing (e.g., recovery of mechanical, electrical, and thermal properties), and assessing the durability of the self-healing capability over multiple cycles [73].

### 5.2. Investigating the Long-Term Durability and Performance After Repeated Self-Repair Cycles

The long-term performance of self-healing CNT materials requires careful consideration of the healing efficiency over numerous cycles. While initial healing might be highly effective, the ability to maintain this level of repair over prolonged use is crucial. Recent studies have demonstrated a remarkable performance over multiple cycles [74,75]. For instance, a CNT/epoxy composite toughened with polycaprolactone (PCL) not only recovered its properties but also exhibited tensile and flexural strength increases of over 15% and 25%, respectively, after a second and third repair cycle under 40% prefabricated damage, with healing efficiencies reaching as high as 116% [63]. Similarly, repair of delaminated carbon/glass composites using resin pre-coating with aminated CNTs restored up to 30% of the compressive strength and significantly increased tensile (19%) and flexural (29%) loads compared to the damaged state [64]. Factors such as the completeness of the repair at the molecular level, demonstrated by the filling of 2–4 µm cracks, and the long-term stability of the doping in functional composites, which has been shown to be stable for over 60 h in some systems, are critical indicators of durability that must be assessed [63,64]. Studies have demonstrated the potential for repeated healing in composite materials. For instance, fiberglass composites with a 3D vascular system have shown autonomous and repeated healing over multiple cycles with high efficiency. Similarly, continuous carbon fiber-reinforced composites with a thermoplastic matrix have been healed numerous times, although with a slight decrease in healing efficiency in subsequent cycles [42]. Epoxy matrix composites have also exhibited self-repair capabilities, but some have shown reduced efficiency after multiple repair cycles [76]. Intrinsic self-healing mechanisms, relying on reversible interactions within the material, offer the potential for numerous healing events, as shown in Table 2. In contrast, extrinsic mechanisms that depend on encapsulated healing agents are limited by the amount of available agent.

The long-term performance of self-healing CNT materials requires careful consideration of the healing efficiency over numerous cycles. While initial healing might be highly effective, the ability to maintain this level of repair over prolonged use is crucial. Factors such as the completeness of the repair at the molecular level, the potential for fatigue of the self-healing mechanism, and the accumulation of byproducts from the repair process can all influence the long-term durability. Self-healing CNT materials intended for use in harsh environments, such as deep space or oceans, will need to demonstrate robustness and the ability to maintain their self-healing capabilities under extreme conditions [25]. Studies on CNT composite elastomers with dynamic bonds have shown promise for long-term stability in applications like electrophysiological signal acquisition [46]. Additionally, certain CNT composites have exhibited good bending durability over a large number of cycles, indicating their potential for long-term performance under mechanical stress [77].

**Figure 3:**
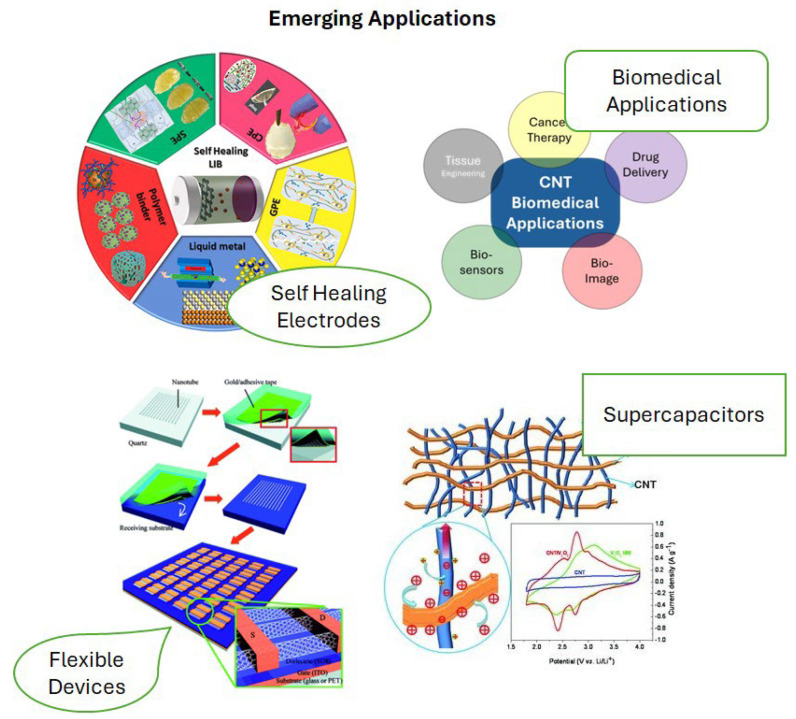
Illustrations for application of CNT self-healing materials, adapted from [82,83,84,85].

The long-term effectiveness of self-healing CNT materials is a critical area for future research. Evaluating the healing efficiency over many cycles, understanding the degradation mechanisms of the self-healing capability, and assessing performance under realistic operating conditions are essential steps towards realizing the full potential of these materials for extended service life and enhanced reliability. Potential applications of these CNT materials are outlined in Table 3 and Figure 3.

## 6. Conclusions

In conclusion, the strategic integration of controlled carbon nanotube (CNT) assembly with self-repair mechanisms represents a frontier in materials science, offering a clear pathway toward creating materials with unprecedented durability and reliability. This review has established that the precise architectural control of CNTs, encompassing their alignment, dispersion, and functionalization, is not merely a method for property enhancement but a foundational prerequisite for the successful implementation of efficient self-repair processes. The synergy between the initial material design and its subsequent ability to heal damage is a central theme that must guide future research and development. Significant progress has been made in developing a diverse array of assembly techniques and both intrinsic and extrinsic self-healing strategies. However, persistent challenges related to the scalability and cost-effectiveness of these methods, as well as achieving perfect, uniform CNT arrangements, currently limit their widespread industrial adoption. Overcoming these engineering hurdles is critical for translating laboratory innovations into real-world applications. The future of self-healing materials hinges on moving beyond phenomenological observations to a deep, quantitative understanding of the molecular-level repair processes. This necessitates the broader adoption and development of advanced, in situ characterization techniques, such as high-powered spectroscopy (XPS, NEXAFS, EELS, AES) and quantitative Raman mapping. These tools are essential for validating healing mechanisms, optimizing material chemistry, and providing the rigorous data needed to build predictive models for material lifetime and performance. Future research should be directed toward creating multifunctional materials that combine self-healing with other intelligent capabilities, such as self-sensing for damage detection or integrated energy storage. By fostering interdisciplinary collaboration and leveraging the unique versatility of CNTs to enable and enhance self-repair, the scientific community can unlock the full potential of these systems. Ultimately, the synergy between CNT assembly and self-repair, validated by advanced characterization, holds immense promise for creating a new generation of innovative, adaptive materials capable of transforming sectors ranging from aerospace and energy to medicine and consumer electronics.

## Figures and Tables

**Table 1:** Advanced spectroscopic techniques for characterization of self-healing in CNT composites.

Technique	Acronym	Information Provided	Application in Self-Healing Analysis	Advantages/Limitations
Raman Spectroscopy	-	Vibrational modes, molecular structure, defect density (ID/IG ratio), stress/strain state.	In situ monitoring of polymerization kinetics; quantitative mapping of defect healing; assessing stress transfer at healed interface.	Non-destructive, high spatial resolution/Signal can be weak, with fluorescence interference.
X-ray Photoelectron Spectroscopy	XPS	Elemental composition, chemical state, C-F/C-O/C-N bonding environments.	Verifying CNT functionalization; characterizing surface chemistry of fractured and healed surfaces.	Quantitative, high chemical specificity/Surface sensitive (~10 nm), requires vacuum.
Near-Edge X-ray Absorption Fine Structure	NEXAFS	Unoccupied electronic states, bond hybridization (sp2/sp3), molecular orientation.	Confirming covalent bond formation during functionalization or healing; probing polymer chain alignment at interfaces.	Sensitive to bonding and orientation/Requires synchrotron source.
Electron Energy Loss Spectroscopy	EELS	Elemental composition, electronic structure (plasmons, C K-edge), bonding state.	Atomic-resolution analysis of defects induced by irradiation; characterizing bonding across the healed interface in a TEM.	Very high spatial resolution/Requires electron-transparent samples, potential beam damage.
Auger Electron Spectroscopy	AES	Elemental composition, chemical state (from C KVV lineshape).	Characterizing the “fingerprint” of carbon bonding (graphitic vs. disordered) at surfaces before/after damage and healing.	Extremely surface sensitive (~1–3 nm)/Can be destructive, requires vacuum.
Soft X-ray Emission Spectroscopy	SXE	Occupied electronic states (valence band).	Provides a complete picture of electronic structural changes when combined with NEXAFS.	Complements NEXAFS/Requires synchrotron source.

**Table 2:** Representative studies of CNT assembly and self-repair.

Reference	Material	CNT Assembly Method	Mechanism	Key
Zamal et al., 2020 [66]	Polymer composite	Dispersion in microcapsules	Polymers	Up to 80% restoration of mechanical and electrical properties.
Gómez-Sánchez et al., 2024 [50]	Epoxy	Homogeneous dispersion	Joule heating of CNT network	Efficient crack recovery with homogeneous resistive heating, healing over 90% of material.
Ouyang et al., 2022 [67]	Carbon fiber polymer	Network in interleaves	Joule heating at interlayer	Delamination repair.
Sambandan, 2012 [68]	Integrated circuits	Dispersion in fluid	Electric-field-induced aggregation	Automated open fault repair triggered by the electric field across the gap.
Wang et al., 2020 [69]	Liquid crystal	Paving aligned sheets	Joule heating of aligned CNT sheets	Healing of scratches with almost no loss of mechanical properties.
Lanzara et al., 2009 [35]	Polymer composite	Nanoreservoirs	Healing agent release from ruptured CNTs	Potential for tougher and automated self-healing materials.
Pu et al., 2018 [70]	Polyurethane composite colydimethylsiloxane (PDMS)	Dispersion	Joule heating via retro-Diels–Alder, polymerization	Crack diagnosing and rapid, repeatable self-healing by electricity or NIR light. Healing time reduced from 24 h to 10 min.
Zhao et al., 2023 [47]	Fiber-reinforced thermoplastic	Embedded film	Joule heating of CNT film	87% self-healing efficiency in terms of flexural strength after low-energy impact.
Gong et al., 2021 [71]	Asphalt binder	Dispersion	Enhanced thermal conductivity for healing	Up to 652% enhancement of mean square displacement at optimal temperatures.

**Table 3:** Examples of applications of self-healing CNT materials.

Application Area	Application Examples	Mechanisms Involved	Reference
Energy Storage	Electrodes in batteries and supercapacitors (flexible, solid-state)	CNT network rearrangement in self-healing polymer matrix	Ibrahim et al., 2025 [49]
Energy Storage	Joule heating for localized repair in epoxy/CNT systems	Thermal activation by the Joule effect	Gómez-Sánchez et al., 2024 [50]
Sensors	Strain and pressure sensors, electronic skin	CNT network sensing and matrix repair; dynamic cross-linking and hydrogen bonding in hydrogels	Del Bosque et al., 2022 [78]
Biomedical	Protective coatings for implants	Extrinsic self-healing using encapsulated agents	Choi et al., 2023 [79]
Biomedical	Drug delivery systems	Potential enhancement of delivery vehicle durability	Zhang et al., 2010 [80]
Aerospace/Automotive	Self-sensing structural components	Potential integration of self-healing for damage repair	Dong et al., 2021 [81]
